# Evaluation of Six Preendoscopy Scoring Systems to Predict Outcomes for Older Adults with Upper Gastrointestinal Bleeding

**DOI:** 10.1155/2022/9334866

**Published:** 2022-01-30

**Authors:** Yajie Li, Qin Lu, Kexuan Wu, Xilong Ou

**Affiliations:** ^1^Department of Gerontology, Zhongda Hospital, School of Medicine, Southeast University, Nanjing, Jiangsu 210009, China; ^2^Department of Gastroenterology, Zhongda Hospital, School of Medicine, Southeast University, Nanjing, Jiangsu 210009, China

## Abstract

**Objectives:**

To compare the ability of six preendoscopic scoring systems (ABC, AIMS65, Glasgow Blatchford score (GBS), MAP(ASH), pRS, and *T*-score) to predict outcomes of upper gastrointestinal bleeding (UGIB) in older adults.

**Methods:**

This was a retrospective study of 602 older adults (age ≥ 65) presenting with UGIB at Zhongda Hospital Southeast University from January 2015 to June 2021. Six scoring systems were used to analyze all patients.

**Results:**

ABC had the largest area under the curve (AUC) (0.833; 95% confidence interval (CI): 0.801–0.862) and was significantly higher than pRS 0.696 (95% CI: 0.658–0.733, *p* < 0.01) and *T*-score 0.667 (95% CI: 0.628–0.704, *p* < 0.01) in predicting mortality. MAP(ASH) (0.783; 95% CI: 0.748–0.815) performs the best in predicting intervention and was similar to GBS, *T*-score, ABC, and AIMS65. The AUCs for MAP(ASH) (0.732; 95% CI: 0.698–0.770), AIMS65 (0.711; 95% CI: 0.672–0.746), and ABC (0.718; 95% CI: 0.680–0.754) were fair for rebleeding, while those of GBS (0.662; 95% CI: 0.617–0.694), *T*-score (0.641; 95% CI: 0.606–0.684), and pRS (0.609; 95% CI: 0.569–0.648) were performed poorly. MAP(ASH) performs the best in predicting ICU admission (0.784; 95% CI: 0.749–0.816). All the five scores were significantly higher than pRS (*p* < 0.05 for ABC, AIMS65 and *T*-score, *p* < 0.01 for GBS and MAP).

**Conclusions:**

Mortality, intervention, rebleeding, and ICU admission in UGIB for older adults can be predicted well using MAP(ASH). ABC is the most accurate for predicting mortality. Except for rebleeding, GBS has an acceptable performance in predicting ICU admission, mortality, and intervention. AIMS65 and *T*-score performed moderately, and pRS may not be suitable for the target cohort.

## 1. Introduction

Upper gastrointestinal bleeding (UGIB) is a common medical emergency. The morbidity is 67–103 per 100000 adults annually [1], and mortality ranges from 2% to 8% [2]. According to epidemiological data, the highest incidence of acute UGIB is in older adults, with about 1% of patients aged 80 years being hospitalized due to acute UGIB [3].

Many risk assessment score systems, including preendoscopy and postendoscopy evaluations, have been developed to predict outcomes such as the need for hospital-based intervention, endoscopic therapy, and admission to an intensive care unit (ICU), rebleeding, and mortality [4]. Some studies showed that these scoring systems distinguish low-risk patients who can potentially be managed as outpatients, allowing more efficient use of resources. Other studies suggested that these score systems distinguish higher-risk patients who might require emergency endoscopy or management in an intensive care unit. The Rockall score and Progetto Nazionale Emorragia digestive score require endoscopy before calculation. However, requiring endoscopy might delay risk assessment in some healthcare units [5]. Some older adults can not tolerate endoscopy. Therefore, recently, investigators have expressed interest in preendoscopic scoring systems for UGIB that can be calculated soon after admission. The most widely established and validated score systems are the preendoscopic Rockall score (pRS), Glasgow Blatchford score (GBS), and AIMS65. Studies showed that the GBS could accurately predict patients who will require intervention; however, its prediction for mortality is relatively poor [6]. Regarding mortality prediction, AIM65 performs better than GBS and pRS; however, the areas under the receiver operator characteristics curves (AUCs of ROCs) are generally no higher than 0.80, suggesting that the clinical application of predicting this endpoint is limited [7]. Several new scoring systems have been developed, including the MAP(ASH) and the ABC scores [8, 9]. Nevertheless, the accuracy of these scoring systems needs to be verified, especially in older adults with UGIB.

This retrospective study compared six preendoscopic risk assessment scores to predict clinically relevant outcomes in older adults. We then determined optimal thresholds for identifying patients at very low risk and could be managed as outpatients and higher-risk patients who might require emergency endoscopy or management in an intensive care unit.

## 2. Methods

### 2.1. Study Design

This was a retrospective cohort study conducted at Zhongda Hospital affiliated with Southeast University from January 2015 to June 2021.

Variceal and nonvariceal UGIB were included in the analysis. Patients were followed for 30 days after discharge. Most patients underwent endoscopy. Only a few patients with poor general conditions did not undergo endoscopy, and they were excluded. The on-duty gastroenterologist determined the timing of endoscopy and whether or not endoscopic therapy was performed.

UGIB was defined as bleeding that develops in the gastrointestinal tract proximal to the ligament of Treitz, presenting with melena or hematemesis [10, 11]. Rebleeding was defined as the melena or hematemesis associated with shock (systolic blood pressure < 100 mmHg, pulse > 100 beats/minute) or decreased hemoglobin concentration greater than 2 g/dL after initially successful treatment [12]. Rebleeding also included cases requiring a second endoscopy, interventional, or radiology surgical intervention.

The indications for blood transfusion were hemoglobin levels decreasing by <7 g/dL in the average patient or <8 g/dL in patients with high-risk heart disease [13]. Endoscopic treatment included diluted epinephrine injection and thermal captive coagulation or clipping. Variceal bleeding was treated by tissue glue injection, band ligation, or transjugular intrahepatic portosystemic shunt.

### 2.2. Data Collection

Patients who presented with melena or hematemesis were included in the analysis. In our research, older adults was defined as aged ≥65 years [14]. Patients aged <65 years or with primary diagnoses other than UGIB were excluded.

We recorded demographic data (age and sex), current medications (antiplatelet drugs, oral anticoagulants, and nonsteroidal anti-inflammatory drugs), comorbidities (chronic pulmonary diseases, cardiac diseases, liver disease, renal disease, disseminated malignancy, hypertension, diabetes, cerebral infarction), clinical presentation, mental state, hemodynamic parameters (pulse rate and blood pressure), hemoglobin, biochemical parameters, including albumin, blood urea nitrogen, and creatinine. We also noted the need for blood transfusion, endoscopic treatment, interventional radiology, surgery, and rebleeding. The clinical outcomes were rebleeding, ICU admission, 30-day mortality, endoscopic treatment, and interventions including transfusion, endoscopic therapy, radiologically guided hemostasis, and surgery.

The data were used to calculate each patient's MAP(ASH), ABC, *T*-score, GBS, pRS, and AIMS65 scores. The methods for calculating four scores (AIMS65, GBS, pRS, and *T*-score) were described previously described [15–17]. Details of the two new scoring systems (ABC and MAP(ASH)) are displayed in Tables 1 and 2.

### 2.3. Data Analysis

We used MedCalc version 19 for statistical calculations. Mean ± standard deviation was calculated for descriptive statistics. ROC curves were used to assess the prognostic value of each scoring system. AUCs of the six scoring systems were calculated one-by-one for mortality, invention, ICU admission, and rebleeding. Then, the AUROCs of the six score systems were compared with one another by using DeLong test. A *p* < 0.05 indicates statistical significance.

## 3. Results

### 3.1. Study Population

A total of 602 older adults with UGIB (age range 65–96 years, mean age 74.0 ± 6.53 years) were retrospectively analyzed. The male/female ratio was 405 : 197.  Table 3 displays patient characteristics, outcomes, and risk scores. 48 patients (8.0%) died within 30 days, and 284 (47.2%) required intervention, while 130 (21.6%) patients suffered from rebleeding.

### 3.2. Comparison among Risk Scores

#### 3.2.1. Mortality

**Figure 1:**
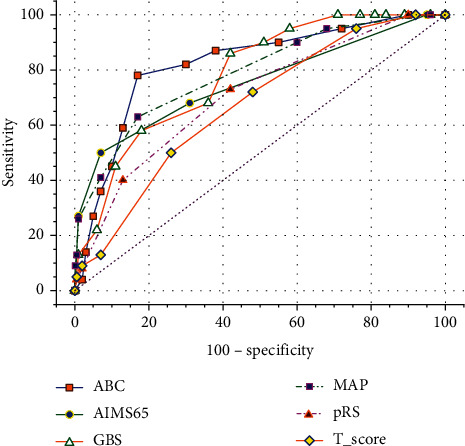
ROC curves for six scoring systems in evaluation of mortality.

The AUCs of the six scoring systems for predicting mortality are listed in Table 4 and Figure 1. ABC had the largest AUC of 0.833 (95% confidence interval (CI): 0.801–0.862) and was significantly higher than that of pRS 0.696 (95% CI: 0.658–0.733, *p* < 0.01) and *T*-score 0.667 (95% CI: 0.628–0.704, *p* < 0.01). The AUCs for MAP(ASH), AIMS65, and GBS were 0.781 (95% CI: 0.746–0.814), 0.754 (95% CI: 0.715–0.792), and 0.755 (95% CI: 0.719–0.789), respectively. There was no significant difference between the three scoring systems and the ABC or pRS scores. The AUCs for MAP(ASH) were significantly higher than that of the *T*-score (*p* < 0.05).

For older adults, the best score cutoffs for predicting 30-day mortality were 6 or more for ABC, 2 or more for AIMS65, 9 or more for GBS, 3 or more for MAP(ASH), 6 or more for pRS, and 9 or less for *T*-score. Using these cutoffs, the sensitivity and specificity were calculated and are displayed in Table 4.

#### 3.2.2. Intervention

**Figure 2:**
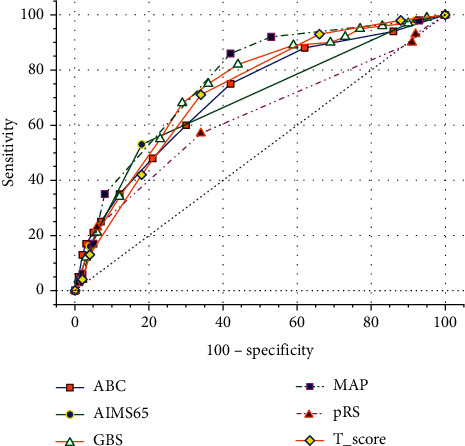
ROC curves for six scoring systems in evaluation of intervention.

The comparisons of the six scoring systems to predict intervention for older adults are shown in Table 5 and Figure 2. The scoring systems in order of largest to smallest AUC were as follows: MAP(ASH)—0.783 (95% CI, 0.748–0.815), GBS—0.749 (95% CI, 0.713–0.783), *T*-score—0.742 (95% CI, 0.705–0.777), ABC—0.718 (95% CI, 0.680–0.754), AIMS65—0.681 (95% CI, 0.642–0.718), and pRS—0.624 (95% CI, 0.586–0.665). MAP(ASH), GBS, *T*-score, ABC, and AIMS65 showed similar effectiveness (*p* > 0.05). The accuracy of pRS for predicting the need for intervention was significantly lower than those of the other five systems (*p* < 0.05 for AIMS65 and *p* < 0.01 for the other four scores).

The best score cutoffs for predicting any intervention were 3 or more for ABC, 1 or more for AIMS65, 8 or more for GBS, 2 or more for MAP(ASH), 3 or more for pRS, and 9 or less for *T*-score.

#### 3.2.3. Rebleeding

**Figure 3:**
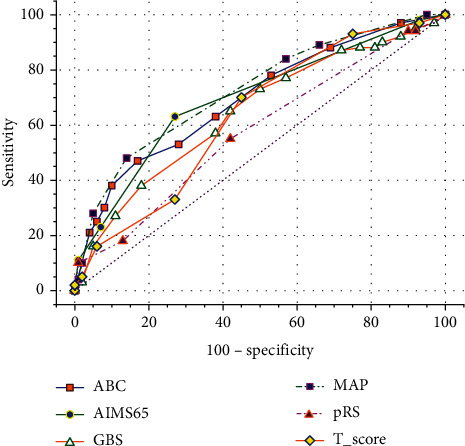
ROC curves for six scoring systems in evaluation of rebleeding.

The comparisons of the abilities of the six scoring systems to predict rebleeding are shown in Table 6 and Figure 3. The scoring systems in order of the largest to smallest AUC were as follows: MAP(ASH)—0.732 (95% CI, 0.698–0.770), ABC—0.718 (95% CI, 0.680–0.754), AIMS65—0.711 (95% CI, 0.672–0.746), GBS—0.662 (95% CI, 0.617–0.694), *T*-score—0.641 (95% CI, 0.606-0.684), and pRS—0.609 (95% CI, 0.569–0.648). The differences between MAP(ASH), AIMS65, ABC, and GBS were not significant (*p* > 0.05). MAP(ASH), ABC, and AIMS65 were more effective than *T*-score (*p* < 0.05) and pRS (*p* < 0.01). The differences between GBS, *T*-score, and pRS were not significant (*p* > 0.05).

The best cutoffs for predicting rebleeding were 6 or more for ABC, 1 or more for AIMS65, 9 or more for GBS, 3 or more for MAP(ASH), 3 or more for pRS, and 9 or less for *T*-score.

#### 3.2.4. ICU Admission

**Figure 4:**
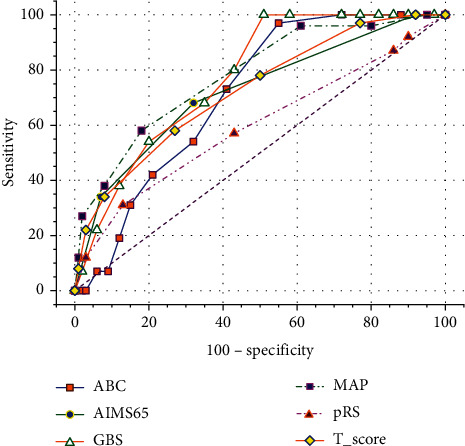
ROC curves for six scoring systems in evaluation of ICU admission.

The comparisons of the ability of the six scoring systems to predict ICU transfer are depicted in Table 7 and Figure 4.The scoring systems in the order of the largest to the smallest AUC were GBS—0.778 (95% CI, 0.743–0.811), MAP(ASH)—0.784 (95% CI, 0.749–0.816), AIMS65—0.730 (95% CI, 0.693–0.765), *T*-score—0.723 (95% CI, 0.685–0.758), ABC—0.711 (95% CI, 0.673–0.747), and pRS—0.600 (95% CI, 0.560–0.639). MAP(ASH), GBS, *T*-score, ABC, and AIMS65 were similarly accurate (*p* > 0.05). All the other five scores were significantly higher than pRS (*p* < 0.05 for AIMS65, ABC, and *T*-score; *p* < 0.01 for GBS and MAP).

The best score cutoffs for predicting ICU admission were 3 or more for ABC, 1 or more for AIMS65, 8 or more for GBS, 3 or more for MAP(ASH), 4 or more for pRS, and 9 or less for *T*-score.

## 4. Discussion

Although its incidence has declined dramatically over the past decade, UGIB remains one of the most common and severe diseases associated with a significant economic burden [18]. For UGIB patients, endoscopy is critical; however, in the world, endoscopy may not be performed timely. Take the UK for example, only 52% of hospitals offer endoscopy during nonworking hours, and only 50% of patients can undergo endoscopy within 24 hours [19]. Also, in most hospitals, the major decisions about patient management are made in the emergency room, where a simple and accurate score is more clinically meaningful to determine whether a patient needs emergency intervention or may avoid admission [8]. Therefore, risk stratification based on clinical risk scores that do not require endoscopy is essential. With the aging population, the incidence of UGIB may increase because the elderly population has a high prevalence of gastroduodenal diseases [19]. Older adults have many complications, their general condition is often poor, and sometimes they can not tolerate endoscopy. In China, the informed consent of family members is required before perform endoscopy. Our research cohort are elderly people, some of whom are old and complicated with various diseases, such as post-PCI, congestive heart failure, chronic lung disease, and chronic renal failure. Endoscopy is risky, and their families may not agree to endoscopic examination. Therefore, the six clinical scoring systems (ABC, AIMS65, GBS, MAP(ASH), pRS, and *T*-score), which are independent of endoscopy, were compared to predict outcomes in older adults with UGIB. In this retrospective study, among 602 older adults, the mortality is like that of a study of older adults in the same district of China [20]. Although the mortality is in the range of previously reported studies [21, 22], it is nevertheless high compared to another study [2].

The ABC score is a newly described preendoscopy risk score based on age, comorbidities, and blood tests [9]. We found that ABC accurately predicted mortality in UGIB and was superior to other UGIB scores, similar to a previous study [9]. Although ABC was not the best-performing scoring system in predicting intervention, ICU admission, rebleeding, and other events, there was no significant difference between ABC and the optimal score in each item (Tables 3–6).

AIMS65 is a simple scoring system involving only clinical observation and biochemical indicators (plasma albumin, international normalized ratio, stress, altered mental state, age, and contraction) [23]. There are few test variables that are easy to remember, and risk classification can be performed without an endoscopic result score, which is suitable for most people. Nevertheless, there are conflicting conclusions about the predictive ability of AIMS65 [24]. In our study, AIMS65 was as good at predicting mortality as the other five scoring systems; however, it was much easier to evaluate. AIMS65, ABC, and MAP(ASH) were equally capable of predicting rebleeding; however, AIMS65 was more accurate than GBS, unlike a previous study [25]. In addition, we found that although AMIS65 was significantly better than pRS in predicting older adults' ICU admission and clinical intervention, it was worse than that of the other four scores for predicting intervention. Since it is not recommended to use the AIMS65 score to grade the risk of rebleeding and other aspects in acute nonvariceal upper gastrointestinal bleeding (ANVUGIB) patients [26], therefore, applying the AIMS65 scoring system requires further research.

The MAP(ASH) score was established in 2020 [8] and includes altered mental status, ASA score, pulse rate, albumin, systolic blood pressure, and hemoglobin. It is a preendoscopic risk score for predicting clinical intervention and predicts the risk of death. According to previous findings [8], MAP(ASH) shows good predictive accuracy for intervention and is fair for mortality. Of the six scoring systems, MAP(ASH) had the highest accuracy in predicting intervention, rebleeding, and the need for ICU admission in our study. MAP(ASH) had the second-highest accuracy in predicting death. MAP(ASH) was superior to the two commonly used scores (GBS and AIMS65). MAP(ASH) is a new score that is simple to calculate and can provide a basis for triage in an emergency department; nevertheless, it still needs to be validated by many clinical studies.

GBS is the most widely used UGIB scoring system with several years of practice and is recommended by several guidelines [26]. In our research cohort, GBS showed the relatively good ability to predict the need for ICU admission and intervention for older adults. Regarding mortality and intervention, GBS was superior to AIMS65, pRS, and *T*-score. However, GBS showed poor performance for rebleeding, which is similar to a previous study [27]. As mentioned in the Asian-Pacific Consensus Group guideline 2018 [28], GBS does not accurately predict rebleeding.

pRS is a simplification of the Rockall score and includes only age, hemodynamics, and complications. It is used for the preendoscopic evaluation of UGIB patients. The accuracy and applicability of the score remain controversial in clinical practice [29]. In the present study, pRS was the worst of the six scores for predicting intervention, rebleeding, and ICU admission. Even for predicting mortality, it was only better than *T*-score. Because the recently updated 2019 international Consensus Group guidelines stated that it could not explicitly recommend or object to the assessment of patients with very low risk of rebleeding or death based on the pRS scores [26], the pRS score for the evaluation of elderly UGIB patients should be used with particular caution.

The *T*-score is a scoring system proposed in 2008 [30] to evaluate the timing of endoscopic examination in patients with UGIB, including the general appearance, pulse rate, number of comorbid diseases, hemoglobin level, and systolic blood pressure. In 2014, a prospective multicenter validation study demonstrated the accuracy of *T*-score in predicting the risk of early endoscopy, rebleeding, and death, similar to GBS [31]. For the cohort in our study, *T*-score did poorly in predicting mortality and rebleeding. For predicting ICU admission, the *T*-score outperformed pRS and ABC; for predicting intervention, it was better than ABC and AIMS65, although without statistical significance, but significantly better than pRS. At present, there are few verifications of this score and a lack of solid evidence for its clinical application [32]. Further verification is therefore required.

In conclusion, older adults with UGIB are more likely to develop severe disease and die during hospitalization. Attention should be paid to appropriate triage and early intervention. To predict mortality, the newly developed ABC score and MAP(ASH) score are the two most appropriate scoring systems, followed by GBS, AIMS65, pRS, and *T*-score. *T*-score showed poor performance (significantly worse than the ABC score), and no significant differences could be found between the other five score systems. MAP(ASH), GBS, *T*-score, and ABC were as effective as each other in predicting intervention for elderly UGIB patients; however, AIMS65 and pRS performed poorly. pRS is significantly worse than the other five scores. MAP(ASH) was the most effective scoring system for rebleeding, followed by ABC and AIMS65. The accuracies of GBS, *T*-score, and pRS were limited for our study cohort. MAP(ASH) and GBS had good predictive accuracy for ICU admission. AIMS65, ABC, and *T*-score were less effective. Again, pRS was the worst. Our findings suggest that, among the current risk scores, the two newly developed scoring systems, especially the MAP(ASH), predict outcomes in older adults with UGIB, and pRS performed poorly in most cases. The main limitation of this study was that the dataset came from only one referral center, and the sample size was not very large. Currently, none of the scoring systems are perfect, and a further work is needed to confirm the effectiveness of these systems.

## Figures and Tables

**Table 1:** The ABC score.

Variable	Value
Age	
60-74years	1
≥75years	2
Blood tests	
Urea > 10 mmol/L	1
Albumin < 30 g/L	2
Creatinine	
100-150 *μ*mol/L	1
>150 *μ*mol/L	2
Comorbidity	
Altered mental status	2
Liver cirrhosis	2
Disseminated malignancy	4
ASA score	
3	1
≥4	3

*ABC*: age, blood tests, and comorbidities; *ASA*: American Society of Anesthesiologists.

**Table 2:** The MAP(ASH) score.

Risk factor	Value
M: altered mental status (Glasgow < 15)	1
A: ASA score > 2	1
P (pulse): HR > 100	1
A: albumin < 2.5 g/dL	2
S: SBP < 90 mmHg	2
H: hemoglobin < 10 g/L	2

*ASA*: American Society of Anesthesiologists; *HR*: heart rate; *SBP*: systolic blood pressure.

**Table 3:** Characteristics of the elderly patients.

Age	74.0 ± 6.53
Sex (male/female)	405 : 197
Comorbidity	
Cirrhosis	68 (10.0%)
Renal failure	60 (9.9%)
Any malignancy	56 (9.3%)
PCI	60 (9.9%)
Heart failure	18 (2.9%)
Hypertension	336 (55.8%)
Diabetes	118 (19.6%)
Chronic lung disease	20 (3.3%)
Medications	
NSAIDs	6 (1.0%)
Aspirin	146 (24.3%)
Clopidogrel	74 (12.3%)
Oral anticoagulants	26 (4.3%)
Steroids	8 (1.3%)
Relevant variables and scores components (median (IQR))	
Systolic blood pressure(mmHg)	124.5 (29)
Pulse (beats/min)	79 (18)
Creatinine (*μ*mol/L)	83 (41)
Hemoglobin (g/L)	88 (43)
Albumin (g/L)	33.7 (8.63)
Urea (mmol/L)	9.65 (9.7)
ASA score	3 (1)
Mental status change	34 (5.6%)
Findings at endoscopy	
Duodenal/gastric ulcer	284 (47.2%)
Erosions	48 (8.0%)
Upper GI cancer	74 (12.3%)
Variceal bleeding	58 (9.6%)
Esophagitis	18 (2.9%)
Mallory-Weiss syndrome	18 (2.9%)
Normal	102 (16.9%)
Outcomes	
Death (total)	48 (8.0%)
Intervention	284 (47.2%)
Rebleeding	130 (21.6%)
Scores (median (IQR))	
AIMS65	1 (1)
GBS	9 (5)
pRS	3 (1)
MAP(ASH)	3 (2)
*T*-score	9 (2)
ABC score	4 (2)

*PCI*: percutaneous coronary intervention; *NSAIDs*: nonsteroidal anti-inflammatory drugs: *IQR*: interquartile range.

**Table 4:** Values of the six scoring systems in prediction of mortality.

Scoring system	Mortality	Cutoff value	Sensitivity, % (95% CI)	Specificity, % (95% CI)
ABC	0.833	6	79.17 (65.0-89.5)	81.95 (78.5-85.1)
AIMS65	0.754	2	43.75 (29.5-58.8)	92.06 (89.5-94.2)
GBS	0.755	9	87.50 (74.8-95.3)	52.71 (48.5-56.9)
Map(ash)	0.781	3	62.50 (47.4-76.0)	81.95 (78.5-85.1)
pRS	0.696	6	66.67 (51.6-79.6)	63.18 (59.0-67.2)
*T*-score	0.667	9	75.00 (60.4-86.4)	50.18 (45.9-54.4)

**Table 5:** Values of the six scoring systems in prediction of intervention.

Scoring system	Intervention	Cutoff value	Sensitivity, % (95% CI)	Specificity, % (95% CI)
ABC	0.718	3	77.46 (72.2-82.2)	58.49 (52.9-64.0)
AIMS65	0.681	1	52.11 (46.1-58.0)	81.13 (76.4-85.3)
GBS	0.749	8	76.76 (71.4-81.5)	63.52 (58.0-68.8)
MAP(ASH)	0.783	2	88.73 (84.5-92.2)	57.86 (52.2-63.4)
pRS	0.624	3	57.04 (51.1-62.9)	66.04 (60.5-71.2)
*T*-score	0.742	9	72.54 (67.0-77.6)	66.67 (61.2-71.8)

**Table 6:** Values of the six scoring systems in prediction of rebleeding.

Scoring system	Rebleeding	Cutoff value	Sensitivity, % (95% CI)	Specificity, % (95% CI)
ABC	0.718	6	41.54 (33.0-50.5)	90.25 (87.2-92.8)
AIMS65	0.711	1	64.62 (55.8-78.0)	73.73 (69.6-77.6)
GBS	0.662	9	63.08 (54.2-71.4)	57.20 (52.6-61.7)
MAP(ASH)	0.732	3	49.23 (40.4-58.1)	86.02 (85.6-89.0)
pRS	0.609	3	58.46 (49.5-67.0)	58.90 (54.3-63.4)
*T*-score	0.641	9	70.77 (62.2-78.4)	53.39 (48.8-58.0)

**Table 7:** Values of the six scoring systems in prediction of ICU admission.

Scoring system	ICU admission	Cutoff value	Sensitivity, % (95% CI)	Specificity, % (95% CI)
ABC	0.711	3	96.15 (86.8-99.5)	45.09 (40.9-49.4)
AIMS65	0.730	1	69.23 (54.9-81.3)	68.73 (64.7-72.6)
GBS	0.778	8	100 (93.2-100)	48.73 (44.5-53.0)
MAP(ASH)	0.784	3	57.69 (43.2-71.3)	81.82 (78.3-85.0)
pRS	0.600	4	30.77 (18.7-45.1)	86.55 (83.4-89.3)
*T*-score	0.723	9	57.69 (43.2-71.3)	72.55 (68.6-76.2)

## Data Availability

The [MedCalc data file] data used to support the findings of this study are available from the first author (Ms. Li Yajie) upon request. Anyone needing the data can contact the following address: Ms. Li Yajie: withlove1982@163.com.

## Abbreviations

UGIB: Upper gastrointestinal bleeding
GBS: Glasgow Blatchford score
pRS: Preendoscopic Rockall score
AUCs: Area under the receiver operator characteristics curves
ICU: Intensive care unit
ASA: American Society of Anesthesiologists
HR: Heart rate
SBP: Systolic blood pressure
PCI: Percutaneous coronary intervention
NSAIDs: Nonsteroidal anti-inflammatory drugs
IQR: Interquartile range.
